# A prototype differential atom interferometer for fundamental physics

**DOI:** 10.1038/s41586-026-10617-1

**Published:** 2026-06-17

**Authors:** C. F. A. Baynham, R. Hobson, O. Buchmüller, D. Evans, L. Hawkins, L. Iannizzotto Venezze, A. Josset, D. Lee, E. Pasatembou, B. E. Sauer, M. R. Tarbutt, T. Walker, O. Ennis, U. Chauhan, A. Brzakalik, S. Dey, S. Hedges, B. Stray, M. Langlois, K. Bongs, T. Hird, S. Lellouch, M. Holynski, B. Bostwick, J. Chen, Z. Eyler, V. Gibson, T. L. Harte, C. C. Hsu, M. Karzazi, C. Lu, B. Millward, J. Mitchell, N. Mouelle, B. Panchumarthi, J. Scheper, U. Schneider, X. Su, Y. Tang, K. Tkalčec, M. Zeuner, S. Zhang, Y. Zhi, L. Badurina, A. Beniwal, D. Blas, J. Carlton, J. Ellis, C. McCabe, G. Parish, D. Pathak Govardhan, V. Vaskonen, T. Bowcock, K. Bridges, A. Carroll, J. Coleman, G. Elertas, S. Hindley, C. Metelko, H. Throssell, J. N. Tinsley, E. Bentine, M. Booth, D. Bortoletto, N. Callaghan, C. Foot, C. Gómez-Monedero, K. Hughes, A. James, T. Leese, A. Lowe, J. March-Russell, J. Sander, J. Schelfhout, I. Shipsey, D. Weatherill, D. Wood, S. N. Balashov, M. G. Bason, K. Hussain, H. Labiad, P. Majewski, A. L. Marchant, D. Newbold, Z. Pan, Z. Tam, T. C. Thornton, T. Valenzuela, M. G. D. van der Grinten, I. Wilmut, K. Clarke, A. Vick, AION Collaboration, C. F. A. Baynham, R. Hobson, O. Buchmüller, D. Evans, L. Hawkins, L. Iannizzotto Venezze, A. Josset, D. Lee, E. Pasatembou, B. E. Sauer, M. R. Tarbutt, T. Walker, O. Ennis, U. Chauhan, A. Brzakalik, S. Dey, T. Hird, S. Lellouch, M. Holynski, J. Chen, Z. Eyler, V. Gibson, T. L. Harte, C. C. Hsu, M. Karzazi, C. Lu, B. Millward, J. Mitchell, N. Mouelle, J. Scheper, U. Schneider, Y. Tang, K. Tkalčec, S. Zhang, J. Carlton, J. Ellis, C. McCabe, G. Parish, D. Pathak Govardhan, T. Bowcock, K. Bridges, A. Carroll, J. Coleman, G. Elertas, S. Hindley, C. Metelko, H. Throssell, J. N. Tinsley, E. Bentine, M. Booth, D. Bortoletto, N. Callaghan, C. Foot, C. Gómez-Monedero, K. Hughes, A. James, T. Leese, A. Lowe, J. March-Russell, J. Sander, J. Schelfhout, I. Shipsey, D. Weatherill, D. Wood, S. N. Balashov, M. G. Bason, K. Hussain, H. Labiad, P. Majewski, A. L. Marchant, D. Newbold, Z. Pan, Z. Tam, T. C. Thornton, T. Valenzuela, I. Wilmut, K. Clarke, A. Vick, S. Hedges, B. Stray, M. Langlois, K. Bongs, B. Bostwick, B. Panchumarthi, X. Su, M. Zeuner, Y. Zhi, L. Badurina, A. Beniwal, D. Blas, V. Vaskonen, M. G. D. van der Grinten

**Affiliations:** 1 Department of Physics, Imperial College London, London, UK; 2 Physics and Astronomy, University of Birmingham, Birmingham, UK; 3 Cavendish Laboratory, University of Cambridge, Cambridge, UK; 4 Physics Department, King’s College London, London, UK; 5 Department of Physics, University of Liverpool, Liverpool, UK; 6 Department of Physics, University of Oxford, Oxford, UK; 7 Rutherford Appleton Laboratory, UKRI-STFC, Didcot, UK; 8 Daresbury Laboratory, STFC, Warrington, UK; 9 Present Address: Nomad Atomics, Richmond, Victoria Australia; 10 Present Address: Jet Propulsion Laboratory, California Institute of Technology, Pasadena, CA USA; 11 Present Address: Institute of Quantum Technologies, German Aerospace Center (DLR), Ulm, Germany; 12 Present Address: Kirchhoff-Institut für Physik, Universität Heidelberg, Heidelberg, Germany; 13 Present Address: Department of Physics & Astronomy, Northwestern University, Evanston, IL USA; 14 Present Address: Physikalisches Institut, Universität Tübingen, Tübingen, Germany; 15 Present Address: Ludwig-Maximilians-Universität München, Munich, Germany; 16 Present Address: Department of Physics, University of Virginia, Charlottesville, VA USA; 17 Present Address: Walter Burke Institute for Theoretical Physics, California Institute of Technology, Pasadena, CA USA; 18 Present Address: Intersect Australia, Sydney, New South Wales Australia; 19 Present Address: Institut de Física d’Altes Energies (IFAE), Barcelona Institute of Science and Technology, Barcelona, Spain; 20 Present Address: Institució Catalana de Recerca i Estudis Avançats (ICREA), Barcelona, Spain; 21 Present Address: Keemilise ja Bioloogilise Füüsika Instituut, Tallinn, Estonia

**Keywords:** Cosmology, Ultracold gases, Laboratory astrophysics, Matter waves and particle beams

## Abstract

Gravitational waves and ultralight dark matter are among the most compelling frontiers in fundamental physics, motivating proposals for very-long-baseline atom interferometerssuch as AION^1^, MAGIS^2^, AICE^3^ and AEDGE^4^ that aim to detect at frequencies at which ground-based^5^ and space-borne^6^ laser interferometers lose sensitivity. Very-long-baseline atom interferometers look for signals by comparing the quantum phase evolution of widely separated atomic ensembles interrogated by a common laser. However, their performance depends critically on suppressing noise sources, particularly laser phase noise. The experimental validation of such noise rejection remains an important challenge. Here we demonstrate a prototype differential atom interferometer based on the single-photon clock transition of fermionic ^87^Sr. Thus, we obtain a gradiometer configuration with a species intrinsically suited to kilometre-scale and space-baseline operation. The instrument operates at the standard quantum limit^7^ with no excess noise beyond atom shot noise. The differential configuration maintains quantum-limited sensitivity in the presence of several radians of artificially injected laser phase noise per shot, which emulates the conditions expected in a very-long-baseline atom interferometer. We also demonstrate the recovery of coherent oscillatory signals across a broad frequency range under fully phase-randomized conditions, a capability that is inaccessible to a single interferometer operating in the same regime. These results provide an experimental validation of the noise-immune measurement principle underlying very-long-baseline atom interferometers and mark an important step towards next-generation quantum sensors for gravitational-wave detection and searches for ultralight dark matter^8,9^.

## Abstract (web summary)

A prototype differential atom interferometer operates at the standard quantum limit with no excess noise beyond atom shot noise, achieving performance in line with the specifications for future long-baseline atom interferometers.

## Main

The discovery of gravitational waves by the LIGO and Virgo laser-interferometer experiments^10^ has opened a new window on the Universe, with prospects for breakthroughs in fundamental physics, astrophysics and cosmology. Just as observations of electromagnetic waves over a wide range of frequencies have provided insights into physical processes within and beyond our Galaxy and in the primordial Universe, it is expected that observing gravitational waves over a wide range of frequencies will offer complementary insights into an equally rich spectrum of phenomena. The operating terrestrial laser-interferometer detectors—LIGO, Virgo and KAGRA—are sensitive to gravitational waves at frequencies around 10^1^ Hz to 10^3^ Hz (refs. ^5,11,12^), and the Laser Interferometer Space Antenna experiment, now under construction, will be most sensitive to gravitational waves with frequencies around 10^−4^ Hz to 10^−1^ Hz (ref. ^6^), leaving unexplored an intermediate range of frequencies around 10^−1^ Hz to 10^1^ Hz.

Important sources of gravitational waves in this frequency range are mergers of intermediate-mass black holes that are heavier than those detected by ground-based laser interferometers and lighter than those targeted by the Laser Interferometer Space Antenna. Such intermediate-mass black holes are thought to be the building blocks for the supermassive black holes^13^ at the hearts of most galaxies, so measurements of their mergers using long-baseline atom interferometers^14,15^ could reveal how supermassive black holes are formed^16^. Further, observations of the slowly evolving inspiral stages of solar-mass mergers would be possible for days or weeks instead of seconds, which would enable multi-messenger astronomy by pinpointing the locations of gravitational-wave sources in the sky^17^.

**Fig. 1:**
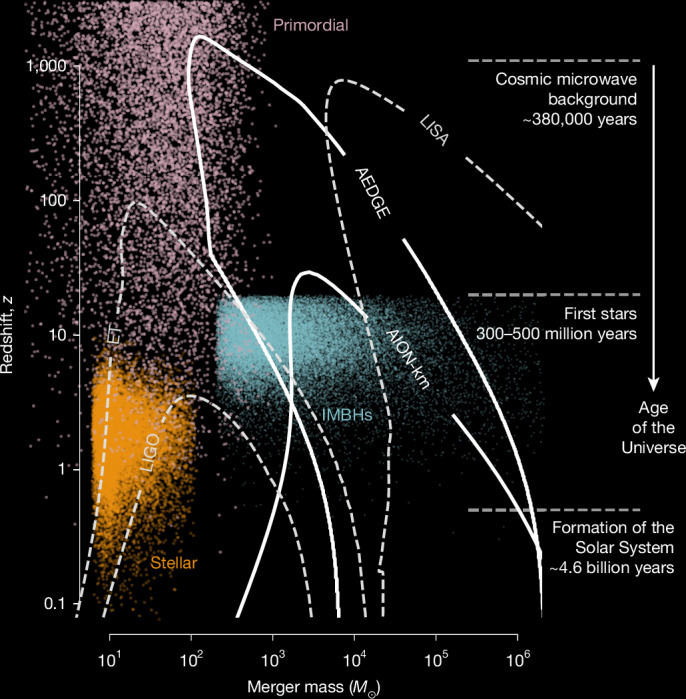
The parameter space of black hole mergers probed by various gravitational-wave detectors, both operational and planned. The horizontal axis gives the mass *M* of the black hole merger causing the gravitational wave, in units of the solar mass. The vertical axis is the distance to the gravitational-wave source, expressed as the redshift *z*. The cyan dots are gravitational-wave signals from a simulation of a 1-year data sample of black hole mergers generated using a hierarchical model of the formation of supermassive black holes^13^, resulting in 6 × 10^4^ simulated events. The orange dots are gravitational-wave signals from a simulated sample of stellar-mass black hole mergers. The violet dots are gravitational-wave signals from a hypothetical population of primordial black holes (see Methods for details). Also shown are the prospective sensitivities of different detectors, including laser-interferometer detectors^5,6,52^ and the AION-km^9^ and AEDGE^4^ atom-interferometer detectors, which have baselines of 1 km and 40,000 km respectively. This figure was inspired by the Cosmic Explorer proposal^52^. IMBHs, intermediate-mass black holes. ET, Einstein Telescope. Source Data

**Fig. 2:**
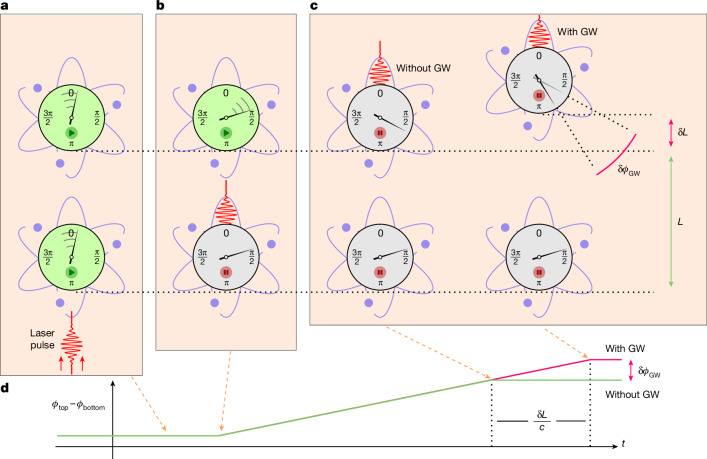
An illustration of the sensitivity of the detector to gravitational waves. **a**–**d**, In the moments before the final π/2 beam-splitter pulse (Fig. 3), the two atom interferometers can be treated as freely falling atomic clocks (**a**) accruing phase at a rate *ω*_0_; the pulse halts this accrual of phase for the lower cloud (**b**), resulting in an accrual of differential phase (**d**) that continues until the pulse reaches the second cloud (**c**). In the proper frame of the bottom cloud (as pictured), the atoms are displaced by a transient gravitational wave (GW). This has the effect of delaying (or hastening) this second interaction, imparting (at leading order) a detectable differential phase of ${\rm{\delta }}{\phi }_{\mathrm{GW}}=\pm \frac{{\rm{\delta }}L}{c}{\omega }_{0}$ (ref. ^36^). **d**, Differential phase accumulated between the two interferometers throughout the sequence, shown with (red) and without (green) a gravitational wave present. Crucially, any phase noise due to the laser pulse itself is strongly suppressed in the differential measurement as it impacts both interferometers equally. The mechanism for sensitivity to dark matter (not pictured) is similar, but results from the modulation of *ω*_0_ instead of *L* (see refs. ^8,40,41,53,54^ for descriptions). This simplified picture neglects complications arising from other interferometer phases *ϕ*_other_ (ref. ^2^), the other pulses in the sequence^19,55^ and the choice of general relativistic gauge^35,36^.

Atom interferometers, which use lasers to split and recombine the wavefunctions of atoms, have optimal sensitivities to gravitational waves with frequencies ${\mathcal{O}}(1)$Hz (refs. ^1,2^) and, hence, are well suited to explore the frequency gap between terrestrial and space-borne laser interferometers, as seen in Fig. 1. With the gradiometer configuration shown in Fig. 2, a differential, single-photon, pair of atom interferometers separated by a baseline *L* of approximately 1 km could have sufficient sensitivity to detect gravitational waves^18,19^ with frequencies of approximately 1 Hz, which, at present, cannot be measured. Such detectors are also sensitive to theorized interactions between atomic constituents and bosonic dark matter fields with masses of approximately 10^−15^ eV (ref. ^8^), with potential resolution significantly beyond that of existing experiments^1^.

Long-baseline atom interferometers are under development by the Atom Interferometer Observatory and Network (AION)^1^ and the Matter-wave Atomic Gradiometer Interferometric Sensor (MAGIS)^2^ collaborations and other projects worldwide^20^. These join other proposed approaches in the mid-frequency band, including space-based laser interferometers such as DECIGO^21^ and magnetically levitated superconducting test masses^22^. See ref. ^23^ for a review. Realizing the potential of atom-interferometer experiments will require overcoming many technical obstacles to reach the target sensitivity. One open question for these projects is whether the laser phase noise, which introduces noise on each individual atom interferometer that is orders of magnitude higher than the standard quantum limit (SQL; Methods), will cancel sufficiently in the gradiometer configuration to reach the SQL. Although the gradiometer principle has previously been demonstrated in experiments using ^88^Sr (ref. ^24^)—or ^87^Rb (refs. ^25,26^), to within known limitations^27^—in this work we quantify the extent of noise cancellation afforded by the scheme. We do this with the more demanding fermionic isotope ^87^Sr, the hyperfine structure and millihertz-linewidth clock transition of which considerably complicate laser cooling and atom interferometry^28–32^. Despite these complications, ^87^Sr is a natural choice for gravitational-wave detection, thanks to its near-ideal properties as an atomic clock isotope^33^ and 150-s excited-state lifetime^34^. These qualities are not shared by other candidate species, such as ^87^Rb or ^88^Sr, but are essential for very-long-baseline experiments, and they enable the extension to space-scale baselines, as proposed by the Atomic Experiment for Dark Matter and Gravity Exploration (AEDGE) project^4^. The same differential measurement configuration that enables gravitational-wave detection with ^87^Sr also provides sensitivity to ultralight dark matter, which would induce coherent oscillations in the clock transition frequency across the detector baseline^8^.

We describe how the AION project has tested a gradiometer configuration in the laboratory using ^87^Sr, combining atomic clock technology with atom interferometry to form two macroscopically separated interferometers interrogated by a common clock laser. Our prototype detector reached the SQL, even in the presence of several radians of synthetic laser phase noise, which emulates the conditions of a full-scale detector. Our results imply laser noise cancellation consistent with full common-mode rejection to within the measurement resolution of our experiment. Finally, we show that the same differential configuration allows the recovery of coherent time-dependent signals, even under conditions where a single interferometer would retain no recoverable phase information. Although further work will be essential to demonstrate laser phase noise cancellation with larger numbers of atoms (for which the SQL is lower) and at longer baselines where the effects of wavefront propagation become relevant, our work verifies the principles underpinning long-baseline, single-photon atom interferometry and passes an important milestone on the road towards measurement of gravitational waves.

Analogously to the interference of light in a laser interferometer, such as that used in the LIGO, Virgo and KAGRA experiments, atom interferometry relies on the interference of quantum matter waves. In the search for gravitational waves, both techniques probe a long baseline whose length in the proper detector frame is modulated by a gravitational wave, which converts the variations in the time of flight of light along this baseline to a variation of the phase in an interference measurement (Fig. 2). For a discussion in a fully relativistic framework, see refs. ^35,36^. In laser interferometers, the interference is between light beams that travel along different paths. In atom interferometers, the interference is between the wavefunctions of atoms that are manipulated by laser pulses to follow spatially separated paths before recombination.

**Fig. 3:**
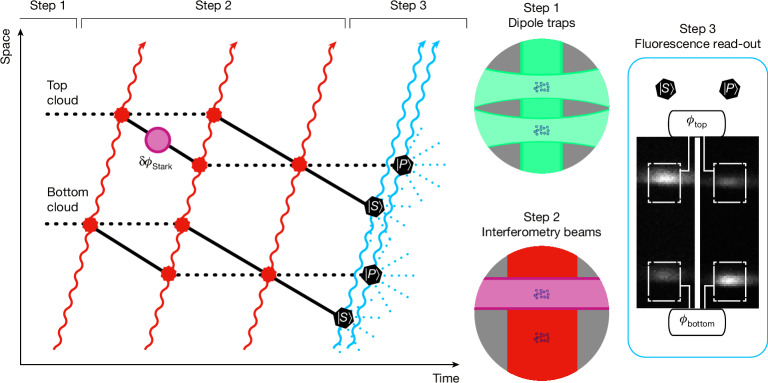
Overview of steps in the experiment. Left, space–time diagram of the paths taken by the arms of each interferometer. Clock pulses (red) create a superposition of the ^1^*S*_0_ (solid lines) and ^3^*P*_0_ (dotted lines) atomic states following both paths. For simplicity, we do not show the free-fall trajectories of the atoms. Right, in step 1, two clouds of ^87^Sr atoms are confined, cooled and then released from crossed optical traps (green). Step 2, an ultrastable clock laser (red) interrogates both clouds. Another beam (purple) applies a phase shift *ϕ*_Stark_ to just the top cloud, inducing a controllable differential phase between the two atom interferometers. Step 3, the interferometer phases are determined by measuring the populations of the ground and excited states using fluorescence measurements. The fluorescence images shown here had an excitation fraction of 20% in the top cloud and 62% in the bottom. See Methods for details of all these steps.

In a single-photon atom interferometer, the atomic wavefunction is manipulated using pulses of light that drive a single-photon transition in the atom, often referred to as a clock transition. For the pulse sequence shown in Fig. 3, the phase of a single interferometer can be written in the simplified form

1 $$\phi ={\int }_{-\infty }^{\infty }{\omega }_{0}\,g(t)\,{\rm{d}}t+{\phi }_{\mathrm{laser}}+{\phi }_{\mathrm{other}},$$

where *ω*_0_ is the angular frequency of the atomic clock transition, *ϕ*_laser_ represents the total phase imprinted on the atoms due to the laser phase during pulses and *ϕ*_other_ comes from various sources, such as static background gravitational or electromagnetic fields^2,19,35,37^, which do not play a role in the dark matter or gravitational wave detection. *g*(*t*) is determined by the relative states of the upper and lower arms of the interferometer:

2 $$g(t)=\left\{\begin{array}{cl}-1, & \mathrm{for}\,t\,\mathrm{between}\,\mathrm{the}\,\text{first beam splitter pulse and}\\  & \text{the mirror pulse},\\ +1, & \mathrm{for}\,t\,\mathrm{between}\,\mathrm{the}\,\text{mirror pulse and the final}\\  & \text{beam splitter pulse},\\ 0, & \mathrm{otherwise}.\end{array}\right.$$

In long-baseline atom interferometry, a fundamental physics signal is extracted by taking the difference in phase δ*ϕ* = *ϕ*_top_ − *ϕ*_bottom_ between two atom interferometers separated by a long distance. To visualize the sensitivity of δ*ϕ* to gravitational waves, the atom interferometers can be conceptualized as atomic clocks that are sensitive to small changes in the time taken for light to traverse the baseline^38^. The clocks ‘tick’ while *g*_top_(*t*) and *g*_bottom_(*t*) are non-zero. These intervals are defined by the arrival times of the light pulse at each interferometer, so a modulation of the baseline *L* by a gravitational wave alters the time counted by the clocks. Alternatively, ultralight dark matter may cause small oscillations in the atomic energy levels, which affect the tick rate *ω*_0_ differently due to the time delay between the two interferometers^8,39–41^. An important technical advantage of taking a differential measurement is that the noise in the laser-induced phase *ϕ*_laser_ cancels in common mode: without laser noise cancellation, it would be unfeasible to achieve the ultimate target phase resolution of 10^−5^ rad/$\sqrt{{\rm{Hz}}}$ in the detector^1^, even when using extremely low-noise lasers (Methods).

Our tabletop prototype of a long-baseline atom-interferometer detector is illustrated in Fig. 3. We operate a pair of crossed optical dipole traps, separated vertically by 1 mm. The traps contain clouds of fermionic ^87^Sr atoms at a temperature of approximately 2 μK loaded from a narrow-linewidth magneto-optical trap (MOT; see Methods for details). When the two clouds are released into free fall, an ultrastable clock laser (described in ref. ^42^) addresses the ^1^*S*_0_ → ^3^*P*_0_ optical clock transition. A first pulse (not shown) selects the slowest atoms from the falling clouds, and a sequence of three pulses then splits, reflects and recombines the selected atoms to create two simultaneous Mach–Zehnder atom interferometers^43^. After the first beam-splitter pulse, we apply another, horizontal laser pulse, off-resonant from the ^1^*S*_0_ → ^3^*P*_1_ transition, to induce a controllable Stark shift *ϕ*_Stark_ to just one of the interferometers (Methods). The same Stark-shifting pulse is applied in every shot of the experiment, which biases the phase offset between the interferometers to aid the data analysis.

**Fig. 4:**
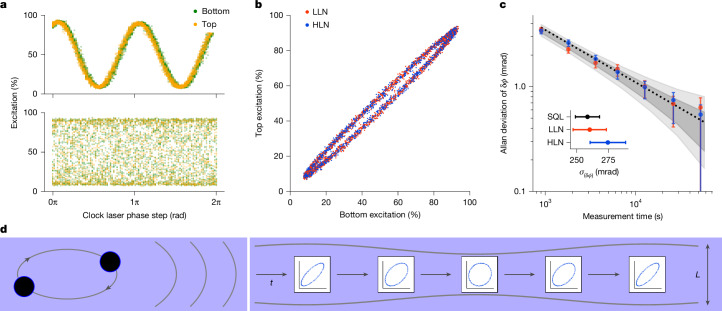
A test of laser noise rejection in the differential-phase measurement. **a**, Clock atom-interferometry fringes in the top and bottom atom clouds with a fixed Stark shift *ϕ*_Stark_ applied to the top cloud. All plots use the same 61.9 h dataset, totalling 56,623 shots. The lower (upper) plot shows the fringes with (without) the addition of artificial laser noise (see Methods for details). **b**, A correlation plot—or Lissajous figure—of the top and bottom atom-interferometer signals, with (blue) and without (red) added laser noise. **c**, Overlapping Allan deviations of the differential-phase time series δ*ϕ*(*t*_*i*_), calculated using unbinned maximum-likelihood estimation. The grey bands represent the 1*σ* and 2*σ* bounds on a SQL prediction from 5,100 Monte Carlo simulations of 56,623 shots each. These were calculated using independently measured experimental inputs, rather than a fit to the Allan deviation data (see Methods). The black dotted line shows the expected averaging behaviour of white noise at the level defined by the theoretical SQL. Inset, the standard errors ${\sigma }_{\langle {\rm{\delta }}\phi \rangle }$ of the differential-phase measurements for the LLN and HLN datasets, compared with the Cramer–Rao theoretical bound (Methods). **d**, An illustration of how a passing low-frequency gravitational wave would modify the shape of the ellipse in the Lissajous figure: a gravitational wave would modulate δ*ϕ* as the second derivative of the strain^19^. Source Data

To gather the datasets presented in Fig. 4, we scanned the relative phases of the three clock pulses applied to both atom interferometers. Figure 4a shows the typical interference fringes that we obtained. Using a π-pulse duration of 44 μs and a free-fall time of *T* = 200 μs between pulses, interferometer contrasts of 0.81 and 0.84 were observed. To simulate the effect of laser phase noise on a long-baseline atom interferometer, we injected randomized phase steps into the clock laser between pulses of the atom-interferometer sequence for one of the datasets. This simulated the effect of a laser phase error that accumulates during the sequence, although it neglects its effect on the fidelity of mirror pulses. This is a reasonable representation of a long-baseline detector, as laser noise will be integrated over drop times of many seconds^1^, thus amplifying its impact relative to our short sequence of 200 μs (see Methods for a calculation). The resulting individual interference fringes are shown in the bottom panel of Fig. 4a. They are completely obscured by the injected noise. However, the differential phase δ*ϕ* of the two interferometers can still be recovered using a maximum-likelihood analysis^44^ applied to the correlated excitation fractions from both interferometers. The likelihood model treats the shot-to-shot common phase as a nuisance parameter, which is marginalized (Methods). A Lissajous correlation plot (Fig. 4b) provides a visualization of the common-mode correlation.

To measure the effect of laser noise on the stability of the differential-phase measurement, we compared measurements with the same applied differential phase *ϕ*_Stark_, but with different levels of applied laser noise. We gathered a low laser noise (LLN) dataset in which only the intrinsic noise of the ultrastable clock laser was present^42^ and a high laser noise (HLN) dataset, which had several radians of laser phase noise artificially added to each shot. The LLN and HLN data were interleaved shot-by-shot, giving a total of 56,623 shots taken over 61.9 h. We extracted from each dataset a time series of the differential phase δ*ϕ*(*t*_*i*_) using an unbinned maximum-likelihood analysis that operated on blocks of 141 excitation measurements from both. In contrast to geometric ellipse fitting of binned Lissajous figures^45^, the maximum-likelihood analysis exhibited negligible bias and reduced statistical error, remaining robust even in the fully phase-randomized regime. Figure 4c shows the Allan deviation^46^ of these datasets; despite laser noise that completely obscures individual interferometer fringes, we observed no statistically significant increase in the differential-phase noise *σ*_δ*ϕ*_ beyond the Cramer–Rao SQL^7,47^ of *σ*_δ*ϕ*_ = 43.5(16) mrad per shot, determined by the 3,100(210) and 2,040(160) atoms measured in the top and bottom traps (Methods). Extrapolating to the full experimental run of 56,623 shots, split between the HLN and LLN datasets, we projected a SQL of ${\sigma }_{\langle {\rm{\delta }}\phi \rangle }=258(10)\,\mathrm{\mu rad}$ in the average differential phase $\langle {\rm{\delta }}\phi \rangle$ for either dataset.

To quantify this rejection, we use the maximum-likelihood analysis to infer the noise levels of the measured δ*ϕ* time series in both cases. Extrapolating to the whole datasets, we determined that the standard deviations ${\sigma }_{\langle {\rm{\delta }}\phi \rangle }$ of δ*ϕ* are consistent with the SQL in both the LLN dataset and the HLN dataset, with ${\sigma }_{\langle {\rm{\delta }}{\phi }_{\mathrm{LLN}}\rangle }-{\sigma }_{\langle {\rm{\delta }}{\phi }_{\mathrm{SQL}}\rangle }=2(16)\,\mathrm{\mu rad}$ and ${\sigma }_{\langle {\rm{\delta }}{\phi }_{\mathrm{HLN}}\rangle }-{\sigma }_{\langle {\rm{\delta }}{\phi }_{\mathrm{SQL}}\rangle }=16(17)\,\mathrm{\mu rad}$ (Fig. 4c, inset). Crucially, we observed no statistically significant increase in noise, despite the addition of several radians of shot-to-shot laser phase noise in the HLN dataset, with ${\sigma }_{\langle {\rm{\delta }}{\phi }_{\mathrm{HLN}}\rangle }-{\sigma }_{\langle {\rm{\delta }}{\phi }_{\mathrm{LLN}}\rangle }=14(19)\,\mathrm{\mu rad}$, consistent with zero additional differential-phase noise within the uncertainty, despite the completely scrambled interferometer phases.

In addition to the Cramer–Rao SQL, we validated the statistical performance of the estimator (bias and coverage) at the SQL using Monte Carlo simulations matched to the experimental conditions (Methods). The Monte Carlo band shown in Fig. 4c is not a fit to the Allan deviation data: it is a prediction constructed from the statistics for the independently measured number of atoms and interferometer contrasts and processed through the same phase-extraction pipeline as the real data. Its agreement with the measured Allan deviation therefore constitutes a non-trivial closure test of the statistical model and is consistent with SQL-limited operation.

Beyond the extraction of a constant differential phase used to quantify the laser noise cancellation, the same maximum-likelihood framework, used in an unbinned mode but with time-dependent δ*ϕ*, enabled hypothesis testing for oscillatory signals in the differential measurement. This provided a proof-of-principle that physically relevant signals—such as those expected from gravitational waves or ultralight dark matter—can be extracted in a differential atom-interferometer configuration under conditions where signal recovery would be impossible using a single interferometer alone. Crucially, this signal-fitting approach remained effective in the shot-to-shot phase-randomized (HLN) regime: a single atom interferometer contained no recoverable phase information in this regime, whereas the differential measurement retained statistically recoverable sensitivity to coherent signals through common-mode noise rejection.

**Fig. 5:**
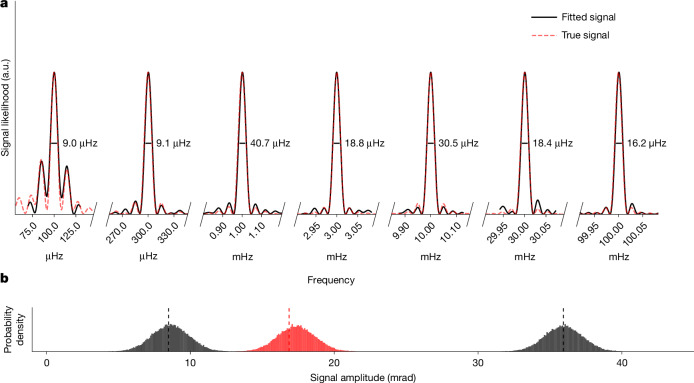
Signal recovery in the presence of HLN. **a**,**b**, An oscillating signal was injected into the differential interferometer to emulate signals from fundamental sources. We extracted maximum-likelihood estimates for the signal frequency *ω*, amplitude *A* and phase *χ* using an unbinned fit over the whole dataset, with all datasets using fully randomized laser phase noise, as described previously, to match conditions in a true long-baseline interferometer. **a**, Signal recovery over seven geometrically spaced frequencies, covering the Nyquist band of our prototype detector. Each injected signal had a nominal amplitude of 17.3 mrad. Solid lines show the likelihood of the presence of a signal extracted from the output of the interferometer. Dotted lines show a periodogram of the true injected signal (artefacts here are due to our finite-length, non-deterministically spaced samples). Despite a dynamic range spanning several orders of magnitude, our detector can resolve injected signals with a ${\mathcal{Q}}$ factor of over 5,000, limited by integration time. Datasets for the 7 signal frequencies from 100 µHz to 100 mHz were 8,603, 13,728, 6,083, 7,262, 6,706, 6,741 and 15,718 shots long, respectively, with a typical sampling rate of 0.3 Hz. **b**, Inferred signal amplitudes for three sample signals. The histograms show probability distributions from Monte Carlo simulations for SQL-limited measurements for the three cases, seeded with their expected amplitudes. The fitted results from the interferometer datasets, marked with dotted lines, are in agreement with our simulated SQL-limited uncertainties. For this test, we used injected signal frequencies of either 1 mHz (black) or 100 mHz (red) to cover the detector range. True datasets were 7,113 and 7,109 shots long for the 1 mHz signals, and 15,718 shots for the 100 mHz data. Simulated distributions used the same durations with 10^6^ repetitions for each dataset. a.u., arbitrary units. Source Data

In Fig. 5, we tested this directly with controlled signal injection and recovery under fully phase-randomized conditions. We injected controlled sinusoidal phase modulations through the off-resonant Stark shift *ϕ*_Stark_ applied to the top interferometer. We analysed the resulting excitation fraction record with the same unbinned likelihood model but applied over the whole dataset. Defining ${\rm{\delta }}\phi (t)={\rm{\delta }}{\phi }_{0}+A\,\sin (\omega t+\chi )$, we demonstrate signal recovery at representative test frequencies spanning the range 10^−4^ Hz to 10^−1^ Hz, with results shown in Fig. 5. These are compared with the signal recovery of a perfect, noiseless detector limited only by integration time, with excellent agreement. These frequencies lie within the measurement bandwidth of the present prototype, set by the shot cycle time of approximately 3 s and the total run duration (hours to approximately 1 day). This choice reflects the prototype operating conditions rather than a fundamental limitation of the method; in a future long-baseline detector, the sensitive band would be shifted by design through the interrogation time, baseline and repetition rate into the mid-frequency regime.

We also tested amplitude recovery by probing signals at a fixed frequency of either 1 mHz or 100 mHz, for various amplitudes (Fig. 5b). We verified that the recovered modulation amplitude scaled linearly with the applied Stark-shift duration and used this nominal calibration to seed 10^6^ Monte Carlo simulations for each scenario to understand the sensitivity of our detector, assuming that there was no noise other than the SQL. All signals were recovered with SQL-limited resolution, and they correctly favoured the null hypothesis when no signal was present.

All signal-injection experiments were performed in the HLN regime, which emulated the behaviour of a long-baseline detector. We found that the differential channel yielded a statistically significant recovery of injected signals, fully rejecting laser phase noise in the signal recovery.

The successful integration of clock transition techniques with atom interferometry is an important milestone on the path towards their joint implementation in quantum sensors with applications in fundamental physics. These include not only the detection of ultralight dark matter and gravitational waves^1,2,9^ but also tests of equivalence principles^48,49^ and measurements of the fine structure constant^50^. The construction of long-baseline detectors will also spur advanced quantum sensing with applications in navigation, geodesy and resource exploration (see, for example, ref. ^26^).

There are many further technical hurdles to be overcome before a long-baseline detector can be realized. These include the development of more intense sources of cold atoms, the extension to longer baselines while controlling the associated systematic shifts^2,37^, large momentum transfer from the laser to the atoms^51^ and the use of squeezed atomic states^47^. All of these are the subjects of R&D programmes in several groups within the international Terrestrial Very-Long-Baseline Atom Interferometry Proto-Collaboration^20^. Nevertheless, the experimental techniques already demonstrated here open up exciting new avenues for scientific exploration that range from probing the fundamental laws that govern our Universe to enhancing quantum sensors.

## Methods

### Cooling sequence

The cold-atom apparatus used in this experiment has previously been described in refs. ^42,56^. To prepare samples of cold ^87^Sr, the atoms are first collected over 1.5 s in a blue three-dimensional MOT that uses the ^1^*S*_0_ → ^1^*P*_1_ transition at 461 nm and a field gradient of 3.5 mT cm^−1^. Atoms that leak into the metastable ^3^*P*_2_ manifold are recycled into the MOT using repump lasers at 679 nm and 707 nm. For efficient repumping of ^87^Sr, frequency sidebands at 585 MHz and 487 MHz are applied to the 707-nm light using an electro-optic modulator to create frequency components near-resonant with transitions from all five hyperfine manifolds of ^3^*P*_2_ (ref. ^57^).

When the blue MOT is switched off, the atoms are captured in a red MOT operating on the ^1^*S*_0_ *F* = 9/2 to ${}^{3}P_{{1}}\,{F}^{{\prime} }=11/2$ transition at 689 nm, using a field gradient of 390 μT cm^−1^. Sidebands at 1,463.265 MHz are applied to the 689-nm light using a resonant electro-optic modulator, such that the *F* = 9/2 to ${F}^{{\prime} }=9/2$ transition stirs the atoms between Zeeman sublevels of the ground state, thus mitigating losses into sublevels where atoms are weakly confined^28^. During the first 220 ms in the red MOT, an intensity of 1,800*I*_sat_ is used for each of the six MOT beams, where *I*_sat_ = 3 μW cm^−2^ is the saturation intensity of the 689-nm transition. To capture the wide range of Doppler-shifted atoms released from the blue MOT, a sawtooth-wave modulation is applied to the 689-nm light at a sweep frequency of 20 nm and a peak-to-peak sweep range of 6 MHz (ref. ^58^). For the following 100 nm, while in the ‘narrowband’ red MOT, the sawtooth frequency modulation is switched off and the intensities of the six MOT beams are ramped linearly from 490*I*_sat_ to 40*I*_sat_. To help support the atoms against the force of gravity, a seventh, unbalanced MOT beam—the ‘up’ beam—is introduced in the vertical direction during the narrowband MOT. The up beam is necessary for creating narrowband red MOTs below 100*I*_sat_ without causing significant atom loss. Upon completion of the narrowband red MOT, the atoms have a temperature of 2 μK and are compressed into a region comparable in size with the optical dipole trap.

### Dipole trap and state preparation

Two crossed optical dipole traps, separated vertically by 1 mm, are formed by separate 2.5-W horizontal beams at 1,064 nm with horizontal and vertical 1/*e*^2^ radii of 220 μm and 23 μm, respectively, crossed with a shared 840-mW vertical beam at 813 nm with 1/*e*^2^ radii of 60 μm in both transverse axes. Overlapping with the top crossed dipole trap, a 4-mW transparency beam at 488 nm, detuned by 25 GHz from the 5*s*5*p* ^3^*P*_1_ → 5*s*5*d* ^3^*D*_2_ transition, is applied with a 1/*e*^2^ radius of 40 μm to protect the atoms from scattered 689-nm light after they are loaded into the top crossed dipole trap region.

Immediately after the free-space red MOT stages described above, the dipole trapping beams, the transparency beam and repumpers at 679 nm and 707 nm are switched on; the red MOT is then held for 100 ms in a ‘top-trap loading’ stage, during which the bias magnetic fields, beam intensities and detunings of the red MOT are optimized to load the atoms into the upper of the two dipole traps. During the top-trap loading stage, the red MOT intensity is linearly ramped from 20*I*_sat_ to 4*I*_sat_ to steadily reduce the atom temperature. Next, to load the bottom optical dipole trap, the red MOT is released for 3 ms by switching off the 689-nm beams. During this time, the cold atoms already in the top trap are held in place, while the hotter atoms fall towards the bottom trap. While the atoms are falling, the vertical bias magnetic field is stepped such that the zero of the quadrupole magnetic field is close to the bottom dipole trap. After 3 ms of free fall, the red MOT beams are switched back on for 100 ms in a ‘bottom-trap loading’ stage using the same parameters as the top-trap loading stage, except for the different bias magnetic field. All but the hottest atoms in the top trap remain in the top trap during the bottom-trap loading stage, as they are protected by the 488-nm transparency beam against scattered 689-nm light.

After both dipole traps are loaded, the MOT beams are switched off, a horizontal bias field is applied and the trapped atoms are optically pumped into the stretched state *M*_*F*_ = 9/2 by applying a horizontal bias field of 38 μT and delivering a 20-ms pulse of circularly polarized light at 689 nm, resonant with the ^1^*S*_0_ *F* = 9/2 to ^3^*P*_1_ ${F}^{{\prime} }$ = 9/2 transition. During the optical pumping, sawtooth-wave frequency modulation is applied to the 689-nm light at a rate of 30 kHz over a range of 6 MHz. Finally, all beams except the dipole trap are switched off, and the bias magnetic field is adiabatically ramped to the final field used for atom interferometry: 31 μT aligned with the linear polarization of the vertical 698-nm clock beam.

### Velocity selection on the clock transition

The clock beam at 698 nm propagates vertically upwards through both dipole trap regions with a waist of 600 μm. The clock laser linewidth is verified against an independent cavity-stabilized laser to ensure that it is below 2 Hz before delivery of the light to atoms through an uncompensated 10-m fibre^42^. Clock spectroscopy sequences are carried out immediately after atoms are released from both dipole traps. The excitation fraction is detected using a 200-μs fluorescence pulse at 461 nm to detect the number of atoms in the ground state ^1^*S*_0_, which is followed by 3.5-ms repumping pulses at 679 nm and 707 nm and another 200-μs fluorescence pulse at 461 nm to detect atoms that are in the ^3^*P*_0_ state after the interferometer sequence. Scattered light from each 461-nm spectroscopy pulse is gathered in separate exposures of an electron-multiplying charge-coupled device (EMCCD) camera (Andor iXon Ultra 897), and a separate EMCCD image without atoms present is used to subtract background counts.

At the maximum available clock power of 640 mW, a Rabi π-pulse time of 44 μs is measured. However, the clock transition was observed to have a peak excitation fraction of 0.3 and a Doppler-broadened linewidth of 60 kHz, which is considerably larger than the 20-kHz Fourier limit. To improve the fidelity of the Rabi pulses in the atom-interferometer sequence, a velocity selection procedure is used. The clock beam is pulsed on for 200 μs at 20 mW, which implements a π pulse that excites the slowest atoms to the upper clock state ^3^*P*_0_. The atoms in the ground state are then pushed away using a 500-μs pulse at 461 nm, leaving only the slow atoms in the ^3^*P*_0_ state to enter the interferometer sequence. After this velocity selection sequence, a resonant, 44-μs Rabi π pulse yielded a peak de-excitation fraction of 90%.

### Clock atom interferometry

The clock atom interferometry consists of a sequence of three resonant pulses on the 698-nm clock transition, with pulse areas π/2 − π − π/2, a π-pulse time *t*_π_ = 44 μs and a dark time *T* = 200 μs between each consecutive pulse. For the data in Fig. 4, the phase of the clock light is always stepped deterministically during the dark times such that the phases of the first, second and third pulses are 0, *ϕ* and 4*ϕ*, respectively, with *ϕ* ranging from 0 to 2π in 100 steps in a randomized order. Each data point in the right-hand side of Fig. 4 is the result of 2 × 100 samples, interleaved between HLN and LLN samples. For the HLN samples, extra phase steps were applied during the interferometer dark times (Fig. 3). The HLN samples were drawn independently from a Gaussian distribution with a standard deviation of 4π rad and mean of 0 rad.

It is important to distinguish between the two types of randomization employed in this work. For both the LLN and HLN datasets, the clock laser phase is scanned deterministically through 100 values in randomized order; this scan-order randomization ensures that any spurious time-oscillatory signals, such as 50 Hz from room lights, are not aliased to look like apparent fringes. For the HLN dataset, we additionally applied large, uncorrelated phase jumps between shots, which fully randomize the absolute phase of each individual interferometer on a shot-by-shot basis. This per-shot phase randomization mimics the regime expected in long-baseline atom interferometers, where integrated laser frequency noise over multi-second interrogation times will produce phase excursions of many radians (see ‘Laser phase noise estimate for a kilometre-scale detector’ section). Under these conditions, a single atom interferometer retains no recoverable phase information, so this provides a stringent test of the noise rejection capability of differential measurements. The phase randomization fully masks the fringes in each individual interferometer but does not affect the measurement of the relative phase of the two interferometers.

### Laser phase noise estimate for a kilometre-scale detector

The phase noise imparted onto the atoms by the laser can generally be calculated from the spectral density of the frequency fluctuations in the laser beam^59^. In our prototype, the laser phase imprinted on each atom interferometer in one repetition of the interferometer sequence beginning at time *t* is approximately *ϕ*_laser_ = *φ*(*t*) − 2*φ*(*t* + *T*) + *φ*(*t* + 2*T*), where *φ*(*t*) is the time-dependent phase of the laser field oscillating as $\cos (kz-{\omega }_{0}t+\varphi (t))$. This approximation holds in the limit of short beam-splitter and mirror pulses separated by a dark time *T* (ref. ^35^). Treating *φ*(*t*) as a stationary noise process with a one-sided power spectral density *S*_*φ*_(*f*) and applying the optical Wiener–Khinchin theorem^60^, we observe a variance in the interferometer laser phase:

$$\begin{array}{l}\langle {\phi }_{\mathrm{laser}}^{2}\rangle \,=\,\langle {(\varphi (t)-2\varphi (t+T)+\varphi (t+2T))}^{2}\rangle \\ \,=\,6\langle \varphi (t)\varphi (t)\rangle -8\langle \varphi (t)\varphi (t+T)\rangle +2\langle \varphi (t)\varphi (t+2T)\rangle \\ \,=\,{\int }_{0}^{\infty }{S}_{\varphi }(f)[6-8\,\cos (2{\rm{\pi }}fT)+2\,\cos (4{\rm{\pi }}fT)]\,{\rm{d}}f.\end{array}$$

For a future long-baseline atom interferometer, we model the clock laser as a thermal-noise-limited, cavity-stabilized laser^61^ with a flicker frequency noise spectrum of the form ${S}_{\varphi }(f)={S}_{\varphi }(f=1\,{\rm{Hz}})\times {(1{\rm{Hz}}/f)}^{3}$. Propagating this functional form through the above equation, the standard deviation of the interferometer laser phase simplifies as $\sqrt{\langle {\phi }_{\mathrm{laser}}^{2}\rangle }=4{\rm{\pi }}T\sqrt{\mathrm{ln}(2)}\sqrt{{S}_{\varphi }(1\,\mathrm{Hz})}$. To provide an optimistic numerical estimate of the laser phase, we assume a laser noise spectrum at the limit of current laser technology, with fractional frequency noise *S*_*y*_(*f*) = (10^−33^/*f*)/Hz (ref. ^62^). For the ^87^Sr clock transition at 698 nm, the corresponding noise spectral density of the clock laser phase fluctuations would be $\sqrt{{S}_{\varphi }(1\,{\rm{Hz}})}$ = 14 mrad/$\sqrt{{\rm{Hz}}}$, resulting in a standard deviation for the interferometer laser phase $\sqrt{\langle {\phi }_{{\rm{laser}}}^{2}\rangle }=710\,{\rm{mrad}}$ for *T* = 5 s, the interferometer time projected for a kilometre-scale detector^1^. Even for an interferometer repetition rate of several shots per second, the laser phase noise imprinted on each individual interferometer is, therefore, far above the level needed to reach the ultimate target phase resolution of $1{0}^{5}\,{\rm{rad}}/\sqrt{{\rm{Hz}}}$ (ref. ^1^), highlighting the need for laser noise cancellation in the differential phase δ*ϕ*.

Compounding the requirements for laser phase noise cancellation, a large momentum transfer of *n* ≈ 10^4^ photon recoils is targeted for long-baseline detectors^1^, which enhances detector sensitivity but imprints laser phase noise *n* times onto each atom interferometer^35^. Taking into account the large momentum transfer, long-baseline interferometers will probably be in the fully phase-randomized regime explored by the HLN dataset in this work.

### Differential bias phase

To induce a consistent relative phase offset between the top and bottom atom interferometers, another, horizontal 689-nm Stark-shifting pulse is applied to only the top interferometer for 30 μs during the gap between the first π/2 pulse and the middle π pulse. The Stark-shifting beam is detuned by −80 MHz from the ^1^*S*_0_ *F* = 9/2 to ^3^*P*_1_ *F*′ = 11/2 transition, with a waist of 500 μm and a power of 1 mW, which induces a phase shift specifically on atoms in the ground state (the lower arm) of the top interferometer. For the data in this paper, the Stark-shifting pulse is used to generate a bias differential phase *ϕ*_Stark_ between the top and bottom interferometers, such that the data lie on a Lissajous ellipse (Fig. 4b) rather than a straight line and, thus, contain more information about the differential phase δ*ϕ*. A non-zero differential bias phase is required for efficient, low-error extraction of δ*ϕ*, whether δ*ϕ* is extracted using a maximum-likelihood estimator or the ellipse-fitting method. In a long-baseline detector, a dark matter or gravitational-wave signal would induce fluctuations in the ellipse fitting angle, on top of the static bias.

### Experimental control

Electronic control signals are produced through the experimental control platform ARTIQ, which uses a field-programmable gate array^63^. The control software is written in Python and is available as open source at ref. ^64^.

### Phase extraction

We extract both constant differential phases (used to quantify laser noise cancellation) and oscillatory-signal components using a unified unbinned maximum-likelihood analysis. For each experimental shot *i*, we modelled the measured excitation fractions (*y*_A,*i*_, *y*_B,*i*_) from the two interferometers A and B as noisy observations of sinusoidal interferometer responses that share a shot-dependent common phase *ϕ*_*i*_ but differ by a differential phase δ*ϕ*(*t*_*i*_). The common phase *ϕ*_*i*_ is treated as a nuisance parameter and marginalized to yield a likelihood that depends only on the differential phase. In practice, we use this marginalized likelihood for inference: we report point estimates from the maximization of the marginal likelihood and compute uncertainties from repeated Monte Carlo simulations performed with matching parameters and analysed using the same analysis pipeline, following a hybrid Bayesian–frequentist approach commonly used in precision measurements and particle physics.

The per-shot likelihood is obtained by numerical integration over the common phase using a uniform prior on [−π, π]:

3 $${{\mathcal{L}}}_{i}={\int }_{-{\rm{\pi }}}^{{\rm{\pi }}}\frac{{\rm{d}}\phi }{2{\rm{\pi }}}\,{\mathcal{N}}(\,{y}_{{\rm{A}},i}| {p}_{{\rm{A}}}(\phi ),{\sigma }_{{\rm{A}},i}^{2})\,(\,{y}_{{\rm{B}},i}| {p}_{{\rm{B}}}(\phi ,{\rm{\delta }}{\phi }_{i}),{\sigma }_{{\rm{B}},i}^{2}),$$

where ${\mathcal{N}}(\cdot | \mu ,{\sigma }^{2})$ denotes a Gaussian probability density. The response functions *p*_A_ and *p*_B_ are sinusoidal fringe models of the form ${p}_{{\rm{A}}}(\phi )={p}_{0,{\rm{A}}}+\frac{{{\mathcal{C}}}_{{\mathcal{A}}}}{2}\cos \,\phi$ and ${p}_{{\rm{B}}}(\phi )={p}_{0,{\rm{B}}}+\frac{{{\mathcal{C}}}_{{\mathcal{B}}}}{2}\cos \,(\phi +{\rm{\delta }}\phi )$, parameterized by offsets *p*_0,{A,B}_ and contrasts ${{\mathcal{C}}}_{\{{\rm{A}},{\rm{B}}\}}$, with noise variance ${\sigma }_{\{{\rm{A}},{\rm{B}}\}}^{2}={p}_{\{{\rm{A}},{\rm{B}}\}}(1-{p}_{\{{\rm{A}},{\rm{B}}\}})/{N}_{\{{\rm{A}},{\rm{B}}\}}$ describing the SQL resulting from the measured *N*_{A, B}_ atoms in the two interferometers. This marginalization enables robust inference, even when individual interferometer fringes are fully washed out by laser phase noise.

#### Mode 1: differential-phase stability analysis

For the stability analysis (Allan deviation) presented in Fig. 4c, we estimated a piecewise constant δ*ϕ* over consecutive blocks of 141 shots.

#### Mode 2: oscillatory-signal analysis

For the oscillatory-signal searches presented in Fig. 5, we parameterized the differential phase as ${\rm{\delta }}\phi (t)={\rm{\delta }}{\phi }_{0}+S\,\sin (\omega t)+C\,\cos (\omega t)$. This parameterization captures the leading-order differential-phase response expected from both gravitational waves and ultralight dark matter fields, which would induce coherent oscillations by modulating the effective light propagation time or the atomic transition frequency. Signal significance is quantified using a likelihood-ratio test statistic that compares the best-fitting model of the signal with the null hypothesis (*C* = *S* = 0). When scanning over frequency, we calibrate the null distribution of the test statistic with Monte Carlo simulations to account for the trials factor. In the absence of an injected signal, the framework correctly favours the null hypothesis. It, thus, provides a statistically well-defined reference for future sensitivity studies. *C* and *S* can be converted to amplitude *A* and phase *χ* using the formulas

$$A=\sqrt{{C}^{2}+{S}^{2}},\,\,\chi =\mathrm{atan}\,2(-C/S).$$

The resolvable frequency band in the prototype is determined by the effective sampling interval in the experiment (set by the average shot cycle time) and the observation duration. At low frequencies, the sensitivity is limited by the finite run duration; at higher frequencies, it is limited by the shot rate and dead time. The injected-signal tests therefore probe the band where the prototype has statistical power over hour-to-day records. In a long-baseline detector, the same analysis framework applies, but the effective response and optimal band are engineered through the interrogation time, repetition rate and baseline to shift the instrumental peak sensitivity into the mid-frequency regime. Accordingly, the resolvable frequency band is instrument-dependent: the frequency band of the prototype implementation does not represent an intrinsic limitation of differential atom interferometry nor of the analysis framework itself.

### Data filtering

The 461-nm, 689-nm and 698-nm laser locks were monitored throughout the experiment. Experiment runs in which one or more of these locks failed or in which the observed number of atoms in either trap was below a manually set threshold near 60% of the median number of atoms were considered invalid and excluded from the data.

### Number of atoms

Atoms are detected at the end of atom-interferometer sequences through fluorescence imaging on an EMCCD camera. Under the assumption that fluorescence scales linearly with the number of atoms, the fluorescence signal can be converted to the number of atoms using a calibration derived from absorption imaging of clouds of atoms prepared under identical conditions as those used for the atom interferometry. The number of atoms *N* in the calibration dataset is extracted from the raw absorption images through the relation *Nσ*(*ω*) = ∫ OD(*x*, *y*) d*x* d*y* (ref. ^65^), where OD(*x*, *y*) is the optical depth of the sample at transverse position (*x*, *y*) in the absorption probe beam and *σ*(*ω*) is the absorption cross section of the ^87^Sr atoms at the laser frequency *ω*.

As the hyperfine shifts of the states ^1^*P*_1_ *F* = 7/2, 9/2 and 11/2 are respectively +37 MHz, −23 MHz and −6 MHz (ref. ^66^), which are significant compared with the 30.5-MHz natural linewidth of the ^1^*P*_1_ state^67^, the absorption cross section *σ*(*ω*) in ^87^Sr generally depends on the polarization and *M*_*F*_. To avoid any reliance on direct measurements of the polarization of our absorption probe light and the *M*_*F*_ state of the atoms, we, instead, measured the absorption amplitudes of the three lines from ^1^*S*_0_ to ^1^*P*_1_* F* = 7/2, 9/2 and 11/2 by carrying out spectroscopy over a ±120-MHz range of detunings using samples of atoms pumped into *M*_*F*_ = 9/2 with the same preparation sequence used for calibrating the number of atoms and for the atom-interferometry datasets. We fitted the peak amplitudes *σ*_7/2_, *σ*_9/2_ and *σ*_11/2_ of the three Lorentzians to the absorption spectroscopy data using fixed literature values for the linewidths and the hyperfine splittings between the Lorentzians^66,67^. Finally, we calibrated the optical depth per unit atom using the identity that the sum of the peak absorption cross sections must match the resonant absorption cross section for the simpler isotopes with zero nuclear spin: ∑_*F*_*σ*_*F*_ = *σ*_0_ = 3*λ*^2^/2π (ref. ^65^). We obtained an uncertainty for the total number of atoms of 8% for the atom-interferometry datasets. This uncertainty is dominated by the uncertainty in the difference in the number of atoms between the calibration dataset and the fluorescence dataset.

For the combined HLN and LLN dataset, the median number of atoms in the top trap was 3,100(210) and in the bottom 2,040(160). The number of atoms in each trap fluctuated during the 61.9 h when the dataset was applied, with a maximum deviation of 15% from the median. No significant difference in the number of atoms was observed between shots with and without induced phase noise.

### Extracting noise levels

To estimate any other form of noise in our measurement of δ*ϕ* caused by injecting laser noise, we applied the maximum-likelihood phase-extraction method independently to both the LLN and HLN datasets. The time series of phases extracted from 141-shot blocks is modelled as

$${\rm{\delta }}\phi ({t}_{i}) \sim {\mathcal{N}}\,({\rm{\delta }}{\phi }_{0},{\sigma }_{{\rm{\delta }}\phi }^{2}),$$

where ${\mathcal{N}}(\mu ,{\sigma }^{2})$ denotes a normal distribution with mean *μ* and standard deviation *σ*. We use a No-U Turn Markov-chain Monte Carlo method implemented in the PyMC package^68^ to sample from the posterior distribution of *σ*_δ*ϕ*_. The mean and 68% credible intervals were *σ*_LLN_ = 3.69(19) mrad and *σ*_HLN_ = 3.89(20) mrad. To compare these with the standard deviations of Monte Carlo simulations with only SQL present (see ‘Monte Carlo SQL’ section), we calculated from these per-block standard deviations the standard error on the mean over the whole dataset, giving ${\sigma }_{\langle {\rm{\delta }}{\phi }_{\mathrm{LLN}}\rangle }=260(13)\,\mathrm{\mu rad}$ and ${\sigma }_{\langle {\rm{\delta }}{\phi }_{\mathrm{HLN}}\rangle }=275(14)\,\mathrm{\mu rad}$.

### Theoretical SQL

We defined the SQL as the Cramer–Rao bound to the per-shot phase noise *σ*_δ*ϕ*_, calculated using the simple likelihood model in equation (3) in which quantum projection noise is the only noise process included. The Cramer–Rao bound is a lower limit to *σ*_δ*ϕ*_ for any unbiased estimator of δ*ϕ*, regardless of the δ*ϕ* extraction technique used. It is used as a rigorous benchmark in differential atom interferometers and quantum sensors^7,47^. For the Cramer–Rao SQL calculation, we differentiate the log-likelihood with respect to variations in δ*ϕ* around a central parameter set, corresponding to the contrasts, number of atoms and mean δ*ϕ* extracted from a maximum-likelihood fit to the full dataset in Fig. 4. We also input the median for the measured number of atoms into the likelihood model. The dominant source of uncertainty in the Cramer–Rao SQL is the approximately 7% uncertainty in the calibration of the number of atoms. We calculated a standard deviation of 43.5(16) mrad per shot. Over the whole dataset of 28,312 shots, this results in a lower bound for the uncertainty in δ*ϕ* of 258(10) μrad.

### Monte Carlo SQL

We validated the unbinned maximum-likelihood phase-extraction method and established the SQL reference using Monte Carlo simulations that replicated the experimental sampling and noise budget. Synthetic shots included the measured contrasts, mean numbers of atoms and their fluctuations, and the projection-noise-limited excitation read-out, with the same estimator applied as in the real data analysis. These tests verified that the estimator was unbiased and that the observed phase variance was consistent with quantum projection noise under the statistics for the measured number of atoms.

**Extended Data Fig. 1:**
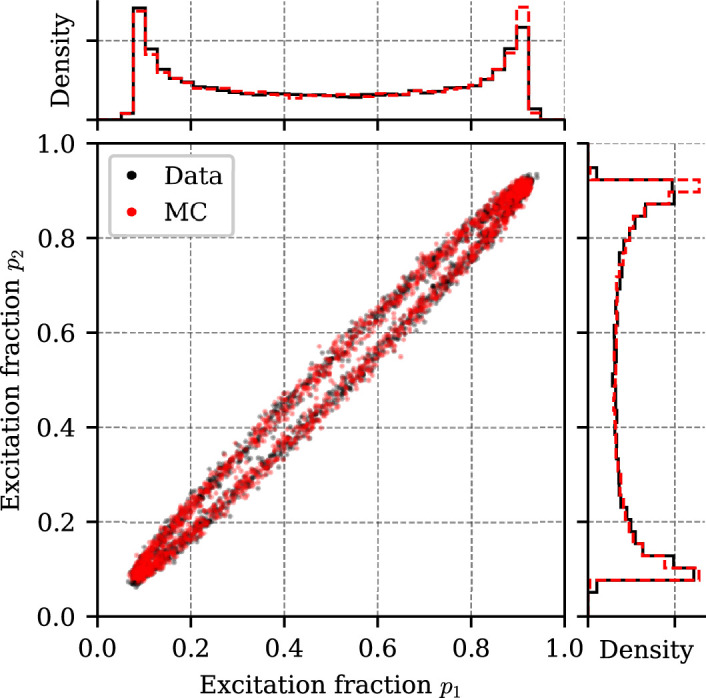
A comparison between Monte-Carlo simulated data with experimental data. Monte-Carlo simulated data contains only quantum projection noise (i.e. the Standard Quantum Limit) as a noise source. The ellipse plot in the *p*_1_, *p*_2_ plane shows 2,000 randomly selected points, whereas the histograms summarise 30,000 true and simulated shots.

We generated 5,100 synthetic datasets such that the variation in the number of atoms was consistent with the uncertainty of the mean from the absorption method described above, the known shot-to-shot variation within datasets and zero other noise sources, as shown in Extended Data Fig. 1. Each dataset consisted of 28,312 simulated interferometer shots, each of which experienced contrasts of 0.81 and 0.84 for the two traps, and the median numbers of atoms were 3,100(210) and 2,040(160), respectively, which matches our real data. We included gaps in the simulated datasets to match the distribution of gaps in our true data. These gaps are caused by various experimental calibrations and outages. We verified that the recovered δ*ϕ* values are unbiased within the statistical uncertainty and that the nominal 68% intervals have the correct frequentist coverage. By calculating an overlapping Allan deviation for each simulation run and then considering the distribution of Allan deviations across all generated datasets, we report the 68% and 95% credible intervals for a differential interferometer limited only by atom shot noise (the SQL), as shown in Fig. 4c. In contrast to the theoretical calculation, the Monte Carlo ensembles reproduce the full experimentally observed distributions for the number of atoms, contrast and projection-noise-limited excitation read-out rather than only their mean values. The resulting SQL reference is, therefore, a prediction analysed with the same estimator as the data.

Statistical compatibility with the SQL prediction was assessed using two complementary tests applied to the Allan deviation in log-space. A global test statistic comparing the measured values with Monte Carlo ensembles at each averaging time yielded *p* = 0.82 for the HLN dataset and *p* = 0.65 for the LLN dataset, indicating that there was no significant deviation from the Monte Carlo SQL prediction. Additionally, the measured Allan deviation slopes (*s* = −0.465 for HLN and *s* = −0.463 for LLN) are consistent with the Monte Carlo SQL ensemble, which itself exhibits white-noise scaling (*s* =−0.5), with *p* = 0.45 and *p* = 0.43, respectively.

## Online content

Any methods, additional references, Nature Portfolio reporting summaries, source data, extended data, supplementary information, acknowledgements, peer review information; details of author contributions and competing interests; and statements of data and code availability are available at 10.1038/s41586-026-10617-1.

## Supplementary information

Source Data Fig. 1

Source Data Fig. 4

Source Data Fig. 5a, 5b

## Data Availability

The datasets generated and analysed during the current study are available at Zenodo (10.5281/zenodo.19592551)^69^. The raw data for the results presented in this work are available at Zenodo (10.5281/zenodo.15166669)^64^. The data underlying Figs. 1–5 and Extended Data Fig. 1 are included in this repository.

## Code availability

Experimental control ARTIQ/Python code for the results presented in this work are available at Zenodo (10.5281/zenodo.15166669)^64^.
